# Regulatory module network of basic/helix-loop-helix transcription factors in mouse brain

**DOI:** 10.1186/gb-2007-8-11-r244

**Published:** 2007-11-19

**Authors:** Jing Li, Zijing J Liu, Yuchun C Pan, Qi Liu, Xing Fu, Nigel GF Cooper, Yixue Li, Mengsheng Qiu, Tieliu Shi

**Affiliations:** 1 School of Life Science and Biotechnology, Shanghai Jiao Tong University, Shanghai 200240, China; 2 Department of Anatomical Sciences and Neurobiology, School of Medicine, University of Louisville, Louisville, KY 40292, USA; 3 School of Agriculture and Biology, Shanghai Jiao Tong University, Shanghai 200240, China; 4 Shanghai Information Center for Life Sciences, Chinese Academy of Sciences, Shanghai 200031, China; 5 Bioinformatics Center, Key Lab of Systems Biology, Shanghai Institutes for Biological Sciences, Chinese Academy of Sciences, Shanghai 200031, China; 6 Daqing Institute of Biotechnology, Northeast Forestry University, Daqing, Heilongjiang 163316, China

## Abstract

**Background:**

The basic/helix-loop-helix (bHLH) proteins are important components of the transcriptional regulatory network, controlling a variety of biological processes, especially the development of the central nervous system. Until now, reports describing the regulatory network of the bHLH transcription factor (TF) family have been scarce. In order to understand the regulatory mechanisms of bHLH TFs in mouse brain, we inferred their regulatory network from genome-wide gene expression profiles with the module networks method.

**Results:**

A regulatory network comprising 15 important bHLH TFs and 153 target genes was constructed. The network was divided into 28 modules based on expression profiles. A regulatory-motif search shows the complexity and diversity of the network. In addition, 26 cooperative bHLH TF pairs were also detected in the network. This cooperation suggests possible physical interactions or genetic regulation between TFs. Interestingly, some TFs in the network regulate more than one module. A novel cross-repression between Neurod6 and Hey2 was identified, which may control various functions in different brain regions. The presence of TF binding sites (TFBSs) in the promoter regions of their target genes validates more than 70% of TF-target gene pairs of the network. Literature mining provides additional support for five modules. More importantly, the regulatory relationships among selected key components are all validated in mutant mice.

**Conclusion:**

Our network is reliable and very informative for understanding the role of bHLH TFs in mouse brain development and function. It provides a framework for future experimental analyses.

## Abstract 2

A comprehensive regulatory module network of 15 bHLH transcription factors over 150 target genes in mouse brain has been constructed.

## Background

Transcription factors (TFs) play pivotal roles in brain development by controlling the sequential generation of neurons and glia from uncommitted progenitor cells [1]. However, little is known about how gene expression programs are differentially unfolded in various cell types. Recognition of specific promoter sequences by transcriptional regulatory proteins is one of the first steps in the initiation of gene expression programs [2-4]. Genome-wide expression profiles provide important information about the transcriptional regulation of various cellular and molecular processes. The basic/helix-loop-helix (bHLH) proteins comprise a large TF family involved in the regulation of a variety of biological processes, including cell proliferation, specification and differentiation during neurogenesis [5]. The bHLH TFs are abundantly expressed in the developing mouse brain [6], and many subfamilies of bHLH proteins, such as the HES, OLIG, NPAS and NEUROD families, have been demonstrated to play crucial roles in the development of the central nervous system [7-11]. The bHLH domain has two functionally distinct regions, the basic region and the HLH region. The DNA-binding basic region at the amino terminus of the bHLH domain (approximately 15 amino acids) has a high content of basic residues, whereas the carboxy-terminal HLH region is formed by two amphipathic helices separated by a loop region of variable length [12]. bHLH proteins can be subdivided into six distinct groups (A to F) in the animal system [5,13]. Briefly, group A proteins bind to the E-box (CAGCTG) and have a distinctive pattern of amino acids (XRX) at sites 5, 8, and 13; group B proteins bind to the G-box (CACGTG) and have a 5-8-13 configuration of K/H-X-R; group C comprises bHLH proteins that have the PAS domain, which bind to non-E-box sites (NACGTG or NGCGTG); group D proteins lack the DNA-binding basic region; group E proteins contain a carboxy-terminal WRPW peptide that preferentially bind to N-boxes (CACGCG or CACGAG); and group F comprises COE-bHLH proteins [5,13,14].

At present, the increasing gene-expression profiles in public databases provide us with opportunities to elucidate the possible transcriptional regulatory networks. Since the whole regulatory network that controls mouse brain function is too complex to be fully understood at the current time, we chose to focus on the bHLH TFs and their related regulatory network, which have been shown to play important roles in mouse brain development. A module network of bHLH TFs was constructed from mining of genome-wide gene expression data and partially validated experimentally. This module network may provide an initial platform for the future study of transcriptional regulation of bHLH TFs in the development and function of mouse brain.

## Results

### Construction of the regulatory network

The module networks procedure identifies modules of co-regulated genes, their regulators and the conditions under which regulation occurs [15]. To construct the module network and understand the regulatory mechanisms of bHLH TF in mouse brain, we inferred a regulatory network from the gene expression data with the module networks method proposed by Segal *et al*. [15].

**Figure 1:**
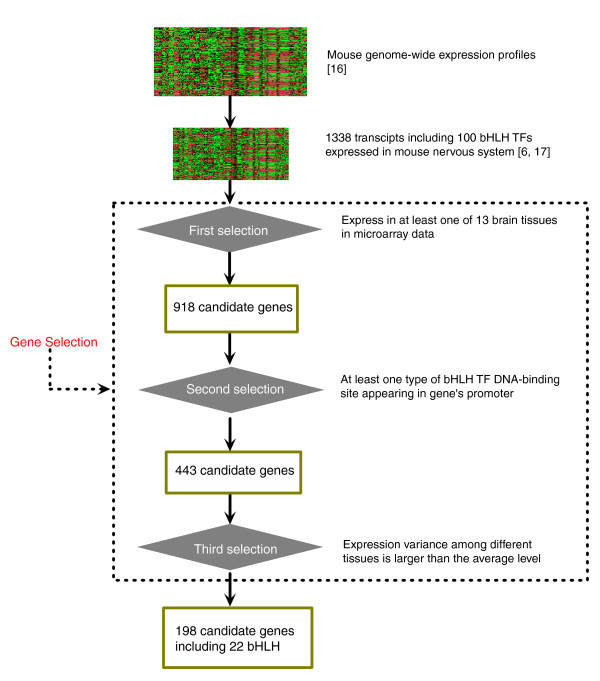
Overview of the gene selection process prior to the construction of the module network.

To provide a convincing and inclusive network, 1,338 transcripts from the mouse genome, including 100 bHLH TFs, were chosen as original candidate genes for constructing a regulatory network from the genome-wide normalized gene expression data [16], all of which have been proven to be expressed in the mouse nervous system by gene cloning and other expression assays [6,17,18]. As shown in Figure 1, we selected 918 genes involving 61 bHLH TFs from the 1,338 candidate genes in the first selection step, which were detected in at least one of 11 mouse brain tissues according to the expression data [16]. These brain tissues included cerebellum, substantia nigra, hypothalamus, frontal cortex, cerebral cortex, dorsal striatum, hippocampus, olfactory bulb, trigeminal, dorsal root ganglia and pituitary. At the beginning, we tried to detect the interactions among different TF families, but obtained unstable results since the number of microarrays was limited to 22. Therefore, we decided to focus on the regulatory relationships between the bHLH TF family and their targets.

**Figure 2:**
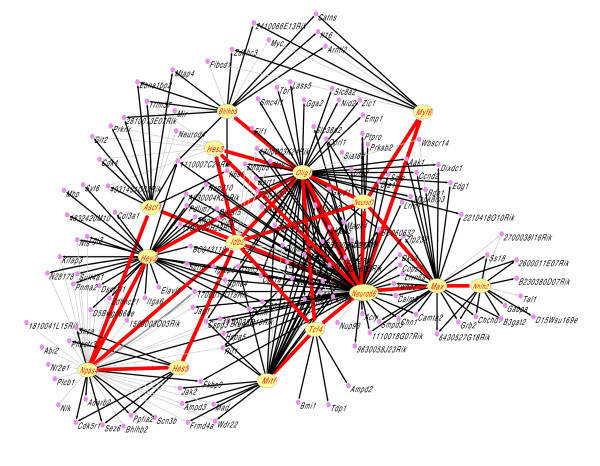
The bHLH regulatory TF network in mouse brain. The graph depicts the inferred regulatory network of bHLH TFs (yellow ellipses) and their target genes (pink dots). Directed lines represent regulation relationship. Directed black connections between a regulator and its target gene are supported by the match analysis of DNA-binding sites. The regulatory relationship between transcription factors is shown by directed red connections.

It is well known that recognition of binding sites (BSs) by TFs is a prerequisite for the initiation of gene expression. Therefore, the promoter sequences of the 857 candidate target genes (excluding the bHLH TFs) were extracted from the PromoSer database [19], including 1,000 bp upstream and 50 bp downstream of each transcription start site. Of the 857 genes, 443 contained one or more reported BSs for bHLH proteins and were further analyzed together with 61 bHLH TFs in the second gene selection step (Figure 1). Here, BSs included both the preferred BSs (E-box, G-box, non-E-box, N-box) of the bHLH proteins of A to F groups and the experimentally confirmed BSs (TRANSFAC Professional 9.3) of bHLH proteins. In the final selection process, both target genes and TFs with expression levels below the average among the different brain tissues were excluded and this yielded the final subset of 198 genes (Figure 1). This gene subset included 22 bHLH TFs and was used to build a regulatory network of bHLH TFs in mouse brain. As a result, the regulatory connections among 153 target genes and 15 bHLH TFs were discovered by the module network approach. The remaining genes, 23 target genes and seven bHLH TFs, were not considered here because no regulatory link among them was detected. With the aid of the Pajek 1.15 program, a hierarchical scale-free network describing the regulations between TFs and their target genes was drawn (Figure 2); this consists of 168 nodes (genes) and 339 directed connections. The nodes represent TFs or their target genes, whereas the connections represent regulatory interactions. Every TF node has a large number of connections with its target genes. The average number of target genes for each TF is 22, with many target genes shared by more than one TF.

In the learned network, 26 coregulating TF pairs were also detected. The hierarchical relationships between the TFs are shown with red lines (Figure 2). Most common transcriptional regulatory motifs described previously were found in the connections between TFs [20]. For example, Olig1-Hey2-Npas4-Ascl1 constitutes a regulatory chain, and Olig1-Hey2-Npas4-Idb2-Olig1 is a multi-component loop. Neurod6 forms a single input structure by regulating Neurod1, Olig1, Myf6, Hes3 and Tcf4. We found that only a few steps are necessary to join any two TFs. This presumably facilitates the efficient propagation and integration of signals [21].

**Figure 3:**
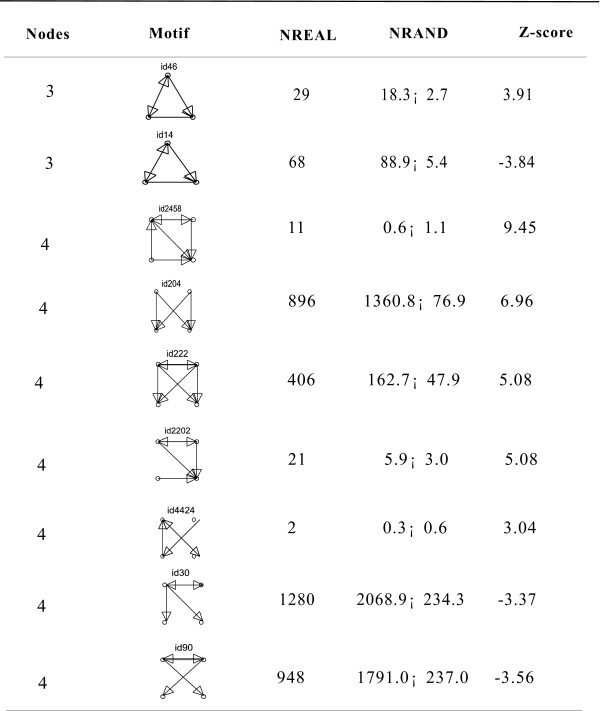
Comparison of the real network with randomized networks. We applied a Z-score to quantify the difference of the network motif between our regulatory network and 100 random networks. The motifs with Z-score greater than 3 or less than -3 are listed in Figure 3. Here, Nodes is the subgraph size; Motifs means subgraphs of the motif [22]; NREAL is the number of a motif in the real network; and NRAND is the average number of a motif in 100 randomized networks.

For the most basic network motif (regulatory pattern), three-node and four-node motifs were detected with mfinder 1.2 in the complete regulatory network [22]. Higher-order motifs were too complex and not detected here. Six distinct three-node motifs and 66 four-node motifs were detected in the network. We applied a Z-score to quantify differences between the network motifs of our regulatory network and 100 random networks. The motifs with a Z-score greater than 3 or less than -3 are listed in Figure 3. The distribution of two three-node motifs and seven four-node motifs in our network are significantly different from their randomized counterparts. The network motifs describe how a single node is connected with its neighbours and demonstrate the complexity and diversity of regulatory mechanisms. The network motifs, in particular those listed in Figure 3, should play important roles in performing sophisticated biological tasks.

### Modules in the regulatory network

Our regulatory network comprises 28 modules (Table 1 and Additional data file 1), with the number of target genes in each module varying from 1 to 18. It is worth noting that co-regulating TF pairs or groups (more than two members) were also detected in the module network (Table 1). For example, the interaction between Id and Olig, inferred regulators in module 21, have been reported in oligodendroglial differentiation [23]. We analyzed each of the inferred modules with regard to a variety of affiliated data sources and evaluated the validity of their regulatory programs.

**Table 1:** Summary of module analysis

					Regulators					
								
No.	Module*	No. of target genes	Coherence (%)^†^	Significant gene annotations	R1	R2	R3	E^‡^	G^§^	L^¶^
1	Calcium-dependent cell-cell adhesion	12	8		Hey2	Npas4		√	√	
2	Sialyltransferase activity	6	17		Neurod6	Neurod1		√	√	
3	Transition metal ion binding	4	50		Neurod6	Max		√	√	
4	Monocyte differentiation	2	50		Tcf4			√	√	
5	Endoplasmic reticulum	7	29		Npas4	Neurod6		√	√	
6	Protein heterodimerization activity	2	50		Npas4					
7	Eye development (sensu) vertebrata)	6	33		Npas4	Heatr1		√	√	
8	Neurotransmitter metabolism	7	14		Hes5	Npas4		√	√	
9	Anion channel activity	1	100		Npas4	Neurod6		√	√	
10	Protein kinase activator activity	3	33		Hey2	Neurod6		√	√	
11	Cation antiporter activity	5	20		Olig1			√	√	
12	Cell surface receptor linked signal transduction	5	60		Neurod6	Max		√	√	
13	Regulation of cell proliferation	6	33		Hes3	Ascl1		√	√	
14	Stem cell division and DNA repair	2	50		Tcf4			√	√	
15	Cellular morphogenesis	5	60	*P *< 0.05	Neurod6	Hey2		√	√	
16	Sequence-specific DNA binding	8	25		Olig1			√	√	
17	Lipid biosynthesis	12	25	*P *< 0.05	Neurod6			√	√	
18	Cytoskeletal regulatory protein binding	6	17		Ascl1	Bhlhb5		√	√	
19	Negative regulation of metabolism	18	17		Olig1	Neurod6	Mitf	√	√	√
20	Monovalent inorganic cation transporter activity	4	25		Nhlh2			√	√	
21	Intracellular non-membrane-bound organelle	6	50		Mitf	Npas4		√	√	√
22	Ribosome	8	13		Olig1	Max		√	√	√
23	Calcium ion binding	9	33		Hey2			√	√	√
24	Menstrual cycle	7	14		Max	Nhlh2		√	√	
25	Cytokine activity	7	14		Bhlhb5	Myf6		√	√	√
26	Endosome	12	8		Olig1	Neurod6	Idb2	√	√	
27	Morphogenesis of embryonic epithelium	5	20		Hey2	Neurod6		√	√	
28	Carboxylic ester hydrolase activity	2	50		Npas4				√	

*Each module was assigned a name based on the smallest *P *value for enrichment of GO categories of genes in the module. ^†^GO coherence of each module, measured as the percentage of genes in the module covered by the category with the smallest *P *value. ^‡^E, experimental evidence showing at least one of the genes in the module is regulated by, or interacts with, the respective TF or the relationship between the TF and its target was proved by the match with an experimentally confirmed DBM. ^§^G, TF-target pair was supported by the match with grouping-DBM in the promoter sequence of genes in the module. ^¶^L, literature data mining provided support for the relationship between a TF and its target gene.

### Module nomenclature

To name the modules and investigate their molecular function, we calculated the hypergeometric functional enrichment score among the modules (Table 1) based on the Gene Ontology (GO) database [24]. Only two modules represent functional enrichments of the utmost significance (Benjaminni correction, *P *< 0.05). Most of the modules identified here are too small to represent significant functional enrichments. Diversity of molecular functions within these modules suggests, for example, that Neurod6 and Hey2 are TFs that modulate a wide spectrum of genes with diverse functions. Each module was assigned a specific name based on the most enriched (with the lowest *P *value) GO categories at layer 5. The GO coherence of each module was measured to determine the percentage of genes in the module covered by the GO category with the lowest *P *value (Table 1). For example, module 15 is regulated by the co-regulating TFs Neurod6 and Hey2 and is here named Cellular morphogenesis module because cellular morphogenesis is the most significantly enriched GO category in the module (*P *< 0.05). Consistent with the module name, 60% of genes in this module play a role in cellular morphogenesis.

In our constructed module network, a target gene can be clustered into only one module. But some TFs can regulate more than one module under different conditions with the same or different co-regulating TFs. For example, Neurod6 regulates modules 10, 15, and 27 with its co-regulator Hey2, but it also regulates module 2 with another co-regulator, Neurod1. We named these TFs as multiple-module (MM) regulators. Npas4 and Neurod6 are representatives of MM regulators, regulating 8 and 11 modules, respectively (Additional data file 1).

### Modules controlled by MM regulators Neurod6 and Hey2

**Figure 4:**
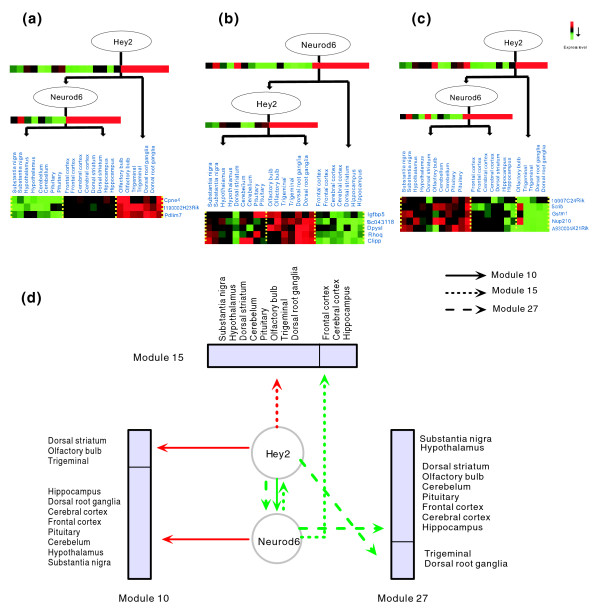
Diagrammatic representation of three modules regulated by Neurod6 and Hey2. **(a-c) **Expression profiles of genes in modules 10, 15, and 27 regulated by Neurod6 and Hey2. Each node in the tree represents a regulator (Hey2 or Neurod6), and the expression of the regulators themselves is shown below their respective nodes. Small boxes represent the gene expression profiles in different brain tissues. All arrays at the bottom are the expression of target genes in the module, in which a row denotes a gene and a column denotes a tissue. **(d) **Hey2 and Neurod6 regulate three modules in different ways among 11 brain tissues. Red arrows refer to positive regulation, and green arrows refer to negative regulation.

Another interesting point in our regulatory network is the presence of co-regulating TF pairs. The most active co-regulating pair, Neurod6 and Hey2, simultaneously regulates modules 10, 15, and 27, which display dissimilar expression patterns (Figure 4a–c). Based on the most enriched GO categories, these three modules are involved in protein kinase activator activity, cellular morphogenesis and morphogenesis of embryonic epithelium, respectively. As shown in Figure 4, the expression profiles of these three clusters in brain tissues are different, but all of them are controlled by Neurod6 and Hey2. These results support the previous report that Neurod6 modulates a wide spectrum of genes with diverse functions [25].

The regulatory motifs of these three modules are feed-forward loops, in which the product of one TF gene regulates the expression of a second TF gene, and both factors together regulate the expression of a third gene (target gene) [20]. In these modules, Neurod6 can regulate target gene expression either directly in some tissues or indirectly through first regulating Hey2 expression in other tissues (Figure 4d). Similarly, Hey2 regulates expression of target genes either directly in some regions or indirectly in other regions through regulating Neurod6. Apparently, the mode (positive or negative) and site (tissue) of gene regulation or co-regulation are different in these three modules. The roles of these two TFs could be reversed and their target genes could be altered in different modules (Figure 4d). Interestingly, the regulatory relationships between Hey2 and Neurod6 in three modules are all negatively correlated (Figure 4d). Based on their expression profiles in three modules (Figure 4a–c), the expression of Hey2 is apparently repressed in the frontal cortex, cerebral cortex, hippocampus and dorsal striatum regions where Neurod6 is expressed at a high level. Conversely, Neurod6 is repressed in the olfactory bulb, trigeminal, dorsal root ganglia and pituitary in which Hey2 is induced. Thus, we can clearly observe opposite or complementary patterns of expression for Neurod6 and Hey2 in various brain tissues. This phenomenon prompted us to propose that Neurod6 and Hey2 cross-regulate each other's expression by switching their functions in different brain regions. To confirm our hypothesis, we performed further analyses on their DNA-binding motifs and sequences. It was found that both Hey2 and Neurod6 have a Glu9/Arg12 pair, which has been confirmed by site-directed mutagenesis experiments and crystal structures to constitute the CANNTG recognition motif [26-29]. Moreover, the CANNTG motif is also found in both promoter regions of these two TFs. The cross-repression between Neurod6 and Hey2 has raised the possibility that they bind to the same target genes and their expression is mutually cross-regulated at the same time. As described above, the diversity of co-regulatory relationships between a pair of TFs allows them to have effects on a variety of molecular activities.

### Validity evaluation

It is well known that the binding of a TF to the promoter of its target genes is a proof for the regulatory relationship. Site-directed mutagenesis experiments and the crystal structures of bHLH proteins have shown that the Glu9/Arg12 pair constitutes the CANNTG recognition motif. The critical Glu9 contacts the first CA in the DNA binding motif (DBM), and the role of Arg12 is to fix and stabilize the position of Glu9 [26-29]. Multiple protein sequence alignments with Multalin [30] showed that 12 TFs of the regulatory network have the Glu9/Arg12 pair in the basic region (Additional data file 1), so those proteins should have the CANNTG recognition motif. Moreover, bHLH proteins of different groups have their own DNA binding specificities [5,13]. All TFs in the network were classified into groups from A to F in agreement with the nomenclature and the evolutionary analysis [5,13]. Therefore, the preferred DBMs of the bHLH TFs of different groups could be predicted (Additional data file 1). Here we named the predictive DBMs of the TFs as group-DBMs. In order to validate the relationships between bHLH TFs and their target genes, we performed match analysis with the promoter sequences of the respective target genes using experimentally confirmed DBMs and the group-DBMs of bHLH TFs. The experimentally confirmed DBMs include both that determined using TRANSFAC Professional 9.3 and the CANNTG motif recognized by Glu9/Arg12 pair. The results show that 235 TF-target gene pairs are verified by experimentally confirmed DBMs, and 115 TF-target gene pairs are supported by group-DBMs. In total, 71% of TF-target gene pairs (Figure 2), distributed in most modules (27 of 28) in the network, are validated by the match of BSs in the promoters. However, as indicated in Figure 2, some TFs, such as Neurod6 and Olig1, are highly supported by TFBSs, whereas other TFs, such as Npas4 and Idb2, have little or no support. One reason could be that some TFs, like Idb2, do not bind DNA and instead function by interacting with other TFs [5]. Another possibility could be that the promoter regions of the genes or the DNA-binding preference of the TFs we obtained have not been fully determined.

As described above, 27 modules are supported by the match of BSs. In order to obtain more support information, we performed literature data mining via PubMed from almost 16 million available articles. Literature data mining was used to predict relationships between genes [31]. The concurrence of an inferred regulator and one of its target genes in published abstracts is evident for five of the modules (Table 1). The absence of concurrence of two given genes may only reflect a lack of publications [31].

### Experimental tests

**Figure 5:**
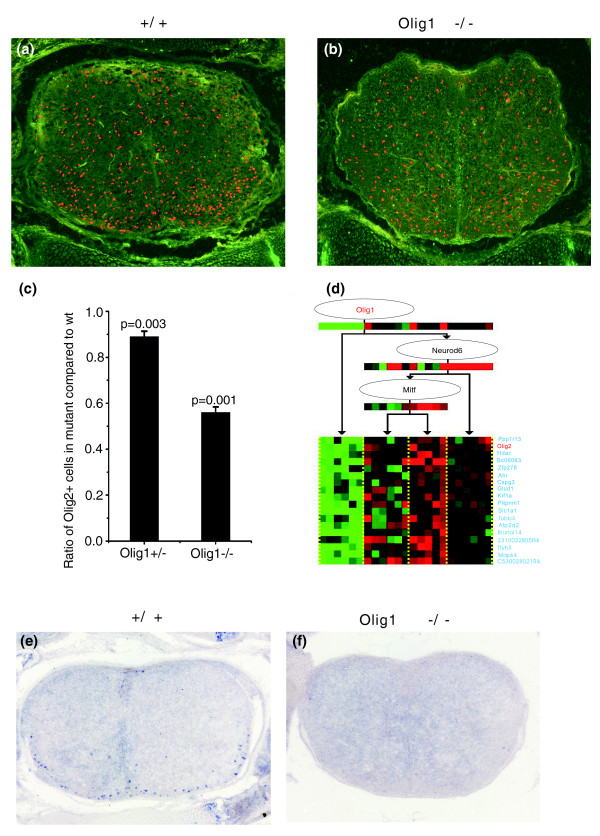
Downregulation of Olig2 and TCF4 expression in Olig1 mutants. Spinal cord sections from E18.5 **(a) **wild-type and **(b) **Olig1 mutant embryos were subjected to immunofluorescence labeling (in red) with anti-Olig2 antibody. The number of Olig2+ cells was significantly reduced in the mutants. **(c) **Statistical analysis of Olig2+ cells in the Olig1+/- and Olig1-/- spinal cords compared to the wild-type (wt). Values were presented as mean ± standard deviation. **(d) **Regulation of the largest module shows that the Olig1 regulates the expression of Olig2. **(e, f) **Spinal cord sections from E18.5 wild-type (e) and Olig1 mutant (f) embryos were subject to *in situ *RNA hybridization with TCF4 antisense riboprobe. Expression of TCF4 was not detected in the mutants at this stage.

Recent studies in the spinal cord showed that Olig1 comprises the combinatorial code for the subtype specification of neurons and glial cells (astrocytes or oligodendrocytes) together with Olig2 [32], which is a target gene of Olig1 in the largest module of the network. The regulatory module (Figure 5d) shows that Olig1 positively regulates Olig2 in different brain tissues. Otherwise, there are both direct (Olig1→Olig2) and indirect regulatory paths (Olig1→Nuerod6→Mitf→Olig2) connecting Olig1 and Olig2. An indirect connection would presumably render Olig2 less sensitive to the inactivation of Olig1while the directed connection would provide more sensitivity.

To experimentally validate the regulatory relationship between Olig1 and Olig2 in the largest module, we examined the expression of Olig2 in the spinal cord of the Olig1 null mutants at embryonic day 18.5. At this stage, Olig1 and Olig2 are primarily expressed in cells of the oligodendrocyte lineage [33-35]. Consistent with the concept that Olig2 is regulated by Olig1, the expression of Olig2 in the mutant spinal cord is significantly reduced (Figure 5a–c). From the results that show that Olig2 is not completely absent in the spinal cord of the Olig1 null mutants, we infer that the regulatory pathway between Olig1 and Olig2 in the spinal cord is indirect. A previous study demonstrated that Olig1 influences Olig2 expression in brain [36]. A recent study indicated that Olig2 influences susceptibility to schizophrenia [37]. As a regulator of Olig2, Olig1 could be considered as another candidate gene for the susceptibility to schizophrenia.

In addition, recent studies showed that both Olig1 and TCF4 (module 26) are expressed in mature oligodendrocytes [38]. In E18.5 mouse embryos, a small number of TCF4-expressing oligodendrocytes could be detected in the wild-type spinal cord sections but not in the mutant spinal cord (Figure 5e, f). This result is consistent with our prediction that Olig1 is a key regulator of TCF4 expression in oligodendrocytes.

**Figure 6:**
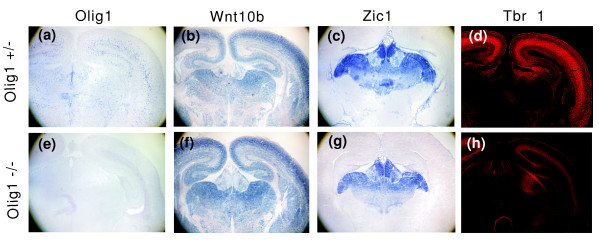
Reduced expression of Zic1 and Tbr1 in Olig1-/- brains. Coronal brain sections at forebrain level from E18.5 **(a-d) **Olig1+/- and **(e-h) **Olig1-/- embryos were subject to *in situ *hybridization with Olig1 riboprobe (a, e), Wnt10b riboprobe (b, f), Zic1 riboprobe (c, g), or immunostaining with anti-Tbr1 (d, h). The expression of Wnt10b was similar between Olig1+/- and Olig1-/- (b, f). The Zic1 expression level was significantly reduced in Olig1-/- thalamus compared to that in Olig1+/- (c, g). Expression of Tbr1 was also reduced in the cortex of Olig1-/- embryos (d, h).

To further test the regulatory relationships between Olig1 and other predicted downstream targets, we compared the expression of Zic1 and Tbr1 (module 11) in embryonic day 18.5 normal and Olig1 mutant brain. In E18.5 wild-type embryos, Zic1 is specifically expressed in the ventral forebrain (Figure 6c), whereas Tbr1 expression is restricted to the cerebral cortex (Figure 6d). Expression of Olig1 was observed in both regions, overlapping with those of Zic1 and Tbr1 (Figure 6a). Consistent with our predicted regulatory relationship, expression of both Zic1 and Tbr1 was downregulated in Olig1-/- mutant brain (Figure 6g, h). In contrast, Wnt10b is not the predicted downstream gene of Olig1, and its expression level in the brain was not affected by the Olig1 mutation (Figure 6b, f).

## Discussion

In this study, we have constructed a transcriptional regulatory network of bHLH TFs in mouse brain using microarray data (gene expression profiles) and the module network method. The Bayesian network method can be used to discover dependency structure between the observed variables, and, therefore, this method is often used as an important approach to infer molecular networks [39]. To some extent, the module network method used in this work can be simply viewed as a Bayesian network in which the variables in the same module share common parameters. Module networks out-perform Bayesian networks even though they are based on the Bayesian network method [15]. Although other approaches for inferring regulatory networks from gene expression data or for identifying modules of co-regulated genes and their shared *cis*-regulatory motifs have been proposed [40-45], the module network can generate detailed testable hypotheses concerning the role of specific regulators and the conditions under which this regulation takes place. Using the same approach, Segal *et al*. [15] accurately identified the module regulatory networks of *S. cerevisiae *with 2,355 genes from 173 microarrays [15]. In the gene-selection process and DBM match analysis, we extracted only a 1,000 bp promoter; however, it is well documented that many neural promoters are much larger than 1 kb. Thus, it is possible that some potential information could have been missed in our analysis.

It is known that many other TF families also play pivotal roles in brain development and it would be interesting and important to study interactions not only within but also between families. However, the amount of public microarray data from brain tissues greatly limits the number of TFs or genes that could be studied in one network. In other words, with limited microarray data, the inclusion of too many genes in a single network could lead to unstable results. So, to maintain the accuracy and robustness of the constructed network, a certain ratio between the number of genes and microarrays should be considered. Considering the limited number of microarrays in this study and the robustness of the potential inferred network, 198 genes with the greatest variance in their levels of expression between different tissues were selected as our final candidate genes in the regulatory network.

Since a relatively small number of TFs from a single family and their target genes are included in this construction, the resulting regulatory network (Figure 2) represents only a small fraction of the whole genome regulatory network in mouse brain. However, even with a limited amount of data this small-scale network can reveal special regulatory features of bHLH TFs.

Most of the modules identified in our network are too small to represent significant functional enrichments. However, the largest module in the network, the Negative regulation of metabolism module (module 19), is composed of three bHLH transcription factors (Olig1, Neurod6, and Mitf) and their 18 target genes (Additional data file 1), whose diverse functions did not lead to function enrichment at a significant level (*P *< 0.05). Although the genes in the network of *Saccharomyces cerevisiae *determined directly from motif occurrences in promoters had better GO coherence [46], the results in this study suggest that genes regulated by the same TFs, and even having similar expression profiles, could have diverse functions. In other words, for target genes, having shared TFs and similar expression patterns does not necessarily indicate that they have the same functions, but instead it suggests that the various functions in the same module are coordinated. Thus, further studies are required to place more emphasis on the functional coordination of these genes.

Our network identified some MM regulators, as represented by Npas4 and Neurod6, suggesting that they could be the core elements of the network and have undoubted regulatory roles in the development of mouse brain. This concept has been substantiated by some recent reports. Npas4 belongs to group C of the bHLH TF family, which features the DNA-recognition motif CACGAG. The Npas4 protein, also called limbic-enhanced PAS protein (LE-PAS) or NXF, was identified in mouse brain tissues independently by two research groups in 2004 [47,48]. At the same time, a novel Npsa4 signaling system was found that may be related to the mental retardation of Down's syndrome [48]. Neurod6, also called Nex, Atoh2 or Math2, is a member of the NEUROD family that is a critical effector of the nerve growth factor pathway and is required *in vivo *for terminal neuronal differentiation [49]. Transcriptional analysis revealed that Neurod6 modulates a wide spectrum of genes with diverse functions, many of which are key downstream regulators of the nerve growth factor pathway and critical to neuritogenesis [25]. Interestingly, the homologs of four target genes of Neurod6 in rat (*Chn1*, *Jag1*, *Glud1 *and *Sort1*) are also found in our regulatory network and are scattered in four modules regulated by Neurod6. The consistency between previous reports and our results provides additional support that the modules detected from our network are tenable.

The cross-repression between the MM regulators Neurod6 and Hey2 was found from the gene expression profiles of three modules. The cross-repression between TFs has been widely identified during embryo development in animals. In the early development stage of vertebrate spinal cord, homeodomain proteins convert a gradient of extracellular Shh signaling activity into discrete progenitor domains through selective cross-repressive interactions between the complementary pairs of class I and class II homeodomain TFs that adjoin the same progenitor domain boundary [50]. In the developing brain, cross-repressive interactions between Otx2 and Gbx2 define the midbrain-hindbrain boundary [51] and interactions between the homeodomain TFs Pax6 and Pax2 help to delineate the diencephalic-midbrain boundary [52].

Cross-repression between transcription factors have also been implicated in regionalization in the embryonic mesoderm [53] and pituitary gland [54]. The same principle has been described during the establishment of anteroposterior polarity within the *Drosophila *embryo [55]. Thus, cross-regulatory interactions between transcription factors appear to be a prevalent strategy for the regional allocation of cell fate. It is possible that the cross-repression of the Neurod6 and Hey2 pair in our network controls various functions related to protein kinase activator activity, cellular morphogenesis and morphogenesis of embryonic epithelium since they are the regulators in those modules. However, the roles of Neurod6 and Hey2 in these biological processes, and how their interactions regulate brain development and specify the different function identities in different brain regions, require further investigations.

## Materials and methods

### Data preparation

Special gene expression profiles in the brain tissues were provided by Su *et al*. [16]. The normalized gene expression data were downloaded from NCBI's Gene Expression Omnibus [56]. We chose the genes that were presented (AP call) in at least one of the following tissues: cerebellum; substantia nigra; hypothalamus; frontal cortex, cerebral cortex; dorsal striatum; hippocampus; olfactory bulb; trigeminal; dorsal root ganglia; and pituitary. All values less than 20 in microarrays were clipped to 20. Log-medium transforms on the data were performed according to the function Y = log2(X/median). To limit the number of gene expression profiles, 198 genes with the greatest variance in their levels of expression between different tissues were identified as candidate genes.

### Construction of network

The software Genomica for creating a module network was downloaded from Weizmann's webpage [57]. A module network was created with default parameters. The whole regulatory network was drawn with Pajek 1.15, which is available from [58,59]. We also used mfinder, a software tool for the detection of network motifs. Its application and source code are available from [60].

### Method to compare a real metabolic network with randomized ones

Following the scheme of Maslov and Sneppen [61], we applied a Z-score to quantify the difference between a real metabolic network and its randomized counterparts:

$$Z=\frac{P-{\bar{P}}_{r}}{\Delta P_{r}}$$

where *P *is the graph metric in the real network, and ${\bar{P}}_{r}$ and Δ*P*_*r *_are the mean and standard deviation, respectively, of the corresponding graph metric in the randomized ensemble.

### Match of DNA-binding motif

The fasta sequences of the promoters, including 1,000 bp upstream and 50 bp downstream of each transcription start site, were extracted from the PromoSer database [62]. The predicted binding sites of genes were obtained according to the categories of TFs from groups A to F with the aid of the existing nomenclature and phylogenetic analysis. Here, an evolutionary tree was built using the neighbor-joining algorithm with MEGA version 3.0 [63]; 1,000 bootstrap replicates were made with the same program to test the statistical reliability. The known DBMs were obtained from the database TRANSFAC Professional 9.3 (updated on 2006.5.30) [64]. Multiple alignments of mouse bHLH protein sequences was performed with Multalin using the default parameters [30,65].

### Enrichment for GO categories in modules

The enrichment for GO categories was analyzed using the tool GOTM [66,67], which reports enrichment (hypergeometric *P *value, Benjaminni correction) with respect to GO categories.

### Literature data mining

Literature data mining was performed with the web-based tool LitMiner [68]. LitMiner is a literature data mining tool that is based on the annotation of key terms in article abstracts present in PubMed [31]. This was followed by statistical co-citation analysis of annotated key terms in order to predict relationships between annotated key terms. Gene names of bHLH TFs in the network were used as key words in the literature data mining.

### Target gene expression studies in Olig1 mutants

The Olig1 mutant mouse line was generously provided by Dr Charles Stiles's Lab at Harvard Medical School. Spinal cord tissues at the thoracic level and brain tissues were isolated from E18.5 mouse embryos and then fixed in 4% paraformaldehyde at 4°C overnight. Following fixation, tissues were transferred to 20% sucrose in phosphate-buffered saline overnight, embedded in Embedding Medium and then sectioned (16 μm thickness) on a cryostat. Adjacent sections from the wild-type and mutant embryos were subsequently subjected to anti-Olig2 and anti-Tbr1 immunofluorescence labeling, or *in situ *RNA hybridization with TCF4, Olig1, Wnt10b and Zic1 riboprobes. *In situ *RNA hybridization and immunofluorescent staining were performed as described previously [69]. Three adjacent spinal cord sections from three independent embryos were immunostained with antibodies. Positive cells containing nuclei in the entire spinal cord sections were counted. Values were presented as mean ± standard deviation. The differences in values were considered to be significant at *P *< 0.05 by Student's *t*-test.

## Abbreviations

bHLH, basic/helix-loop-helix; BS, binding site; DBM, DNA-binding motif; GO, Gene Ontology; MM, multiple module; TF, transcription factors; TFBS, TF binding site.

## Authors' contributions

T Shi and Y Li initiated and directed this research. J Li built the regulatory network of bHLH TFs in mouse brain, made further computational analysis and drafted the manuscript. M Qiu designed the experiments and supervised the process; Z Liu conducted the experiments. Q Liu and X Fu provided assistance in the acquisition of data and revised the manuscript. T Shi, M Qiu, Y Li, Y Pan and N Cooper gave advice and helped in writing the manuscript. All authors read and approved the final manuscript.

## Additional data files

The following additional data are available with the online version of this paper. Additional data file 1 is a table listing summarized information about modules, including module names, TFs and their targets, and support information from different resources.

## Supplementary Material

Additional data file 1 Summarized information about modules, including module names, TFs and their targets, and support information from different resources. Click here for file
