# Supplementary material for: Regulatory module network of basic/helix-loop-helix transcription factors in mouse brain
Source: Genome Biol. 2007 Nov 19;8(11):R244. doi: 10.1186/gb-2007-8-11-r244 (PMC2258200; doi:10.1186/gb-2007-8-11-r244)
Supplement: Additional data file 1 — Summarized information about modules, including module names, TFs and their targets, and support information from different resources. [file gb-2007-8-11-r244-S1.doc]

## Supplemental table1. Summarize information of modules

| **#** | **Module** | **Target gene** | **TF1** | **TF2** | **TF3** |
| --- | --- | --- | --- | --- | --- |
| **1** | calcium-dependent cell-cell adhesion |  | Hey2 | Npas4 |  |
|  |  | Dscr1l1 | E |  |  |
|  |  | 1700018O18Rik | E | E G |  |
|  |  | Kifap3 | E | G |  |
|  |  | 1500003O03Rik | E | G |  |
|  |  | Nts | E |  |  |
|  |  | Sult4a1 | E |  |  |
|  |  | N28178 | E | G |  |
|  |  | D5Bwg0860e | E |  |  |
|  |  | Pnma2 | E |  |  |
|  |  | N28178 | E | G |  |
|  |  | Lgi3 | E |  |  |
|  |  | Zdhhc21 | E | G |  |
| **2** | sialyltransferase activity |  | Neurod6 | Neurod1 |  |
|  |  | Ptpro | E G | E G |  |
|  |  | Max |  |  |  |
|  |  | Myf6 |  |  |  |
|  |  | Siat8d |  |  |  |
|  |  | Oprl1 | E | E |  |
|  |  | Rif1 | E | E |  |
| **3** | transition metal ion binding |  | Neurod6 | Max |  |
|  |  | Lrrn6a | E | E |  |
|  |  | Zfp238 | E G | E G |  |
|  |  | Smpd3 | E | E |  |
|  |  | Chn1 | E G | E G |  |
| **4** | monocyte differentiation |  | Tcf4 |  |  |
|  |  | Ampd2 | E G |  |  |
|  |  | Mitf |  |  |  |
| **5** | endoplasmic reticulum |  | Npas4 | Neurod6 |  |
|  |  | Fkbp9 | E G | E |  |
|  |  | Brunol6 | G | E G |  |
|  |  | 1700018O18Rik | E G | E G |  |
|  |  | Ascl1 |  |  |  |
|  |  | Hspa5 | E G | E G |  |
|  |  | Jag1 | G | E G |  |
|  |  | 1110032O16Rik | G | E G |  |
| **6** | protein heterodimerization activity |  | Npas4 |  |  |
|  |  | Bhlhb2 |  |  |  |
|  |  |  |  |  |  |
| **7** | eye development (sensu Vertebrata) |  | Npas4 |  |  |
|  |  | 1810041L15Rik |  |  |  |
|  |  | Nr2e1 |  |  |  |
|  |  | Adarb2 | E G |  |  |
|  |  | Hes5 |  |  |  |
|  |  | Scn3b | E G |  |  |
|  |  | Abi2 | G |  |  |
| **8** | neurotransmitter metabolism |  | Hes5 | Npas4 |  |
|  |  | Phactr3 | E |  |  |
|  |  | Snca | E | E G |  |
|  |  | Sez6 | E | E G |  |
|  |  | Idb2 |  |  |  |
|  |  | Cdk5r1 | E | E G |  |
|  |  | Ppfia2 | E | E G |  |
| **9** | anion channel activity |  | Npas4 | Neurod6 |  |
|  |  | Ttyh3 | G | E G |  |
| **10** | protein kinase activator activity |  | Hey2 | Neurod6 |  |
|  |  | Cpne4 | E | E |  |
|  |  | 1190002H23Rik | E | E G |  |
|  |  | Pdlim7 | E | E |  |
| **11** | cation antiporter activity |  | Olig1 |  |  |
|  |  | Tbr1 | E M |  |  |
|  |  | Zic1 | E M |  |  |
|  |  | Neurod6 |  |  |  |
|  |  | Bhlhb5 |  |  |  |
|  |  | Slc8a2 | E G |  |  |
| **12** | cell surface receptor linked signal transduction |  | Neurod6 | Max |  |
|  |  | Camta2 | E | E |  |
|  |  | 1110018G07Rik | E G | E |  |
|  |  | Calm3 | E | E G |  |
|  |  | Ywhaz | E | E |  |
|  |  | Dkk3 | E G | E |  |
| **13** | regulation of cell proliferation |  | Hes3 | Ascl1 |  |
|  |  | 4931431C02Rik |  | E |  |
|  |  | Neurod4 |  |  |  |
|  |  | Git2 |  | E |  |
|  |  | Cdk4 |  | E G |  |
|  |  | Trim37 |  | E |  |
|  |  | Prkrir |  | E G |  |
| **14** | stem cell division and DNA repair |  | Tcf4 |  |  |
|  |  | Bmi1 | E G |  |  |
|  |  | Tdp1 | E |  |  |
| **15** | cellular morphogenesis |  | Neurod6 | Hey2 |  |
|  |  | Igfbp5 | E | E |  |
|  |  | BC043118 | E | E |  |
|  |  | Dpysl5 | E | E |  |
|  |  | Rhoq | E | E G |  |
|  |  | Cipp |  |  |  |
| **16** | sequence-specific DNA binding |  | Olig1 |  |  |
|  |  | Cpt1a | E G |  |  |
|  |  | Gga2 | E |  |  |
|  |  | Smc4l1 | E |  |  |
|  |  | Lass5 | E G |  |  |
|  |  | Emp1 | E |  |  |
|  |  | Elf1 | E |  |  |
|  |  | Nid2 | E G |  |  |
|  |  | Hey2 |  |  |  |
| **17** | lipid biosynthesis |  | Neurod6 |  |  |
|  |  | Il6st | E |  |  |
|  |  | Prkab2 | E |  |  |
|  |  | Acly | E |  |  |
|  |  | Dia1 | E G |  |  |
|  |  | Slc38a2 | E |  |  |
|  |  | Lrrn1 | E |  |  |
|  |  | Sqle | E |  |  |
|  |  | Capn2 | E |  |  |
|  |  | Ctsd | E G |  |  |
|  |  | Cops5 | E |  |  |
|  |  | 9630058J23Rik | E |  |  |
|  |  | Nup93 |  |  |  |
| **18** | cytoskeletal regulatory protein binding |  | Ascl1 | Bhlhb5 |  |
|  |  | 1110007C24Rik | E | E |  |
|  |  | Mir |  |  |  |
|  |  | Ebna1bp2 | E | E |  |
|  |  | Mtap4 | E | E |  |
|  |  | Il6st | E | E |  |
|  |  | 2810013E07Rik | E G | E G |  |
|  |  | Trim37 | E | E |  |
| **19** | negative regulation of metabolism |  | Olig1 | Neurod6 | Mitf |
|  |  | Ppp1r13b | E G | E G | E |
|  |  | Olig2 | L M |  |  |
|  |  | Hdac5 | E | E | E |
|  |  | BC060632 | E | E | E G |
|  |  | Zfp278 | G | G | G |
|  |  | Abr |  |  |  |
|  |  | Cspg3 | E G | E G | E G |
|  |  | Glud1 | E | E | E |
|  |  | Kif1a | E G | E G | E |
|  |  | Pitpnm1 | E G | E G | E |
|  |  | Slc1a1 | E G | E G | E |
|  |  | Tubb4 | E | E | E |
|  |  | Atp2a2 | E G | E G | E |
|  |  | Brunol4 | E | E | E |
|  |  | 2310022B05Rik | E | E | E G |
|  |  | Ttyh3 | E G | E G | E G |
|  |  | Mapk4 | E G | E G | E G |
|  |  | C530028O21Rik | E G | E G | E |
| **20** | monovalent inorganic cation transporter activity |  | Nhlh2 |  |  |
|  |  | D15Wsu169e | E G |  |  |
|  |  | B230380D07Rik | E |  |  |
|  |  | Gabpa | E |  |  |
|  |  | Tal1 |  |  |  |
| **21** | intracellular non-membrane-bound organelle |  | Mitf | Npas4 |  |
|  |  | Wdr22 | E | G |  |
|  |  | Ampd3 | E G | G |  |
|  |  | Frmd4a | E |  |  |
|  |  | Rif1 | E |  |  |
|  |  | Jak2 | E | G |  |
|  |  | Mad | EL |  |  |
| **22** | ribosome |  | Olig1 | Max |  |
|  |  | Ccnd2 | E G | E G |  |
|  |  | Aak1 | E G | E |  |
|  |  | Edg1 | E G | E |  |
|  |  | Mapk8ip3 | E | E |  |
|  |  | Wbscr14 |  |  |  |
|  |  | 2210418O10Rik | E G | E |  |
|  |  | Rdh1 | E | EL |  |
|  |  | Dixdc1 | E | E |  |
| **23** | calcium ion binding |  | Hey2 |  |  |
|  |  | 4832420M10 | E |  |  |
|  |  | Elavl2 | E |  |  |
|  |  | Mbp | EL |  |  |
|  |  | Npas4 |  |  |  |
|  |  | Nts | E |  |  |
|  |  | Syt6 | E |  |  |
|  |  | Capn2 | E G |  |  |
|  |  | Itga6 | E |  |  |
|  |  | Col3a1 | E |  |  |
| **24** | menstrual cycle |  | Max | Nhlh2 |  |
|  |  | 2700038I16Rik |  |  |  |
|  |  | Chchd1 | E G | E G |  |
|  |  | 6430527G18Rik | E | E G |  |
|  |  | Grb2 | E | E |  |
|  |  | Nhlh2 |  |  |  |
|  |  | Ss18 | E G | E G |  |
|  |  | 2600011E07Rik | E | E |  |
|  |  | B3gat2 | E G | E |  |
| **25** | cytokine activity |  | Bhlhb5 | Myf6 |  |
|  |  | Il16 | E | E |  |
|  |  | Arntl2 | L |  |  |
|  |  | Catns | E | E |  |
|  |  | Myc |  |  |  |
|  |  | Zdhhc3 | E G | E G |  |
|  |  | 2410066E13Rik | E G | E G |  |
|  |  | Fibcd1 |  |  |  |
| **26** | endosome |  | Olig1 | Neurod6 | Idb2 |
|  |  | Wnt7a | E | E |  |
|  |  | Slc12a5 | E G | E G |  |
|  |  | Ppp1r3c | E G | E G |  |
|  |  | Neurod1 |  |  |  |
|  |  | Tcf4 | M |  |  |
|  |  | Dnajb5 | E G | E G |  |
|  |  | Sort1 | E | E |  |
|  |  | Npy | E | E |  |
|  |  | Vsnl1 | E | E |  |
|  |  | Hes3 |  |  |  |
|  |  | Dnajb5 | E G | E G |  |
|  |  | 1300003K24Rik | E G | E G |  |
| **27** | morphogenesis of embryonic epithelium |  | Hey2 | Neurod6 |  |
|  |  | 1110007C24Rik | E | E |  |
|  |  | Scrib | E | E |  |
|  |  | Gstm1 | E G | E G |  |
|  |  | Nup210 | E | E G |  |
|  |  | A930004K21Rik | E G | E |  |
| **28** | carboxylic ester hydrolase activity |  | Npas4 |  |  |
|  |  | Plcb1 |  |  |  |
|  |  | Nlk | G |  |  |

Columns TF1, TF2 and TF3 show the supporting information of each TF-target pair. E, the support from the match of experiment-confirmed DNA-binding site. G, the support from the match of grouping DNA-binding site. L, the support from literature data mining. M, the regulatory relationship is validated in mutant mice of this study.
